# A commentary on ‘Re-emergence of SARS-CoV-2 Omicron subvariant XBB.1.5: present status, treatment, and future outlook – 2023’

**DOI:** 10.1097/JS9.0000000000000576

**Published:** 2023-06-22

**Authors:** Si-Un Frank Chiu, Jiun-Rung Chen, Chong-Chi Chiu

**Affiliations:** a Department of Computer Science; b Department of Economics, Brown University, Providence, Rhode Island, USA; c Division of Pulmonary and Respiratory Medicine, Department of Internal Medicine; d Department of General Surgery; e Department of Medical Education and Research, E-Da Cancer Hospital; f School of Medicine; g School of Chinese Medicine for Post Baccalaureate, College of Medicine, I-Shou University, Kaohsiung, Taiwan

*Dear Editor,*

The editorial by Akash *et al*.^1^ highlighted the re-emergence and threat of severe acute respiratory syndrome coronavirus 2 (SARS-CoV-2) Omicron subvariant XBB.1.5, which should raise global concerns.

SARS-CoV-2 constitutes the cause of coronavirus disease 2019 (COVID-19). COVID-19 was at one time responsible for an extremely high number of daily infections and deaths. The SARS-CoV-2 infection rates gradually stabilised and passed the threshold of transition into a post-peak period. However, viral diseases that are highly infectious may emerge or re-emerge unexpectedly at various times and regions. Low-income countries or those with fragile health systems are particularly vulnerable to such diseases. Moreover, SARS-CoV-2 has continually mutated, with the Omicron subvariant exhibiting a particular tendency to mutate and exhibiting strong transmission and low pathogenicity. Nevertheless, in the United States, the public health emergency declaration concerning the COVID-19 outbreak expired on 11 May 2023, with other COVID-19-related emergency authorisations also ending. This incongruity between policy and reality is a cause for global concern.

COVID-19’s emergence has negatively affected social interaction, the economy, religion, education, national and international markets, labour, transportation, global stability, and human freedom. Over 450 million people have contracted the disease, with 6 million dying from it^2^. In the past pandemic episodes, people suffered from psychological problems such as paranoia, posttraumatic stress disorder, panic, and anxiety. In other words, pandemic-related stress would engender relatively high burnout in the public. Thus, we suggest that post-peak period interventions should focus on averting COVID-19-induced stress and diminishing burnout.

The pandemic may re-emerge, possibly through SARS-CoV-2 mutating again into a more infectious variant, governments relaxing quarantine- or disease-prevention-related policies, and medical systems becoming disrupted. Bedford *et al*.^3^ anticipated epidemics to be more frequent, severe, difficult to prevent and contain, and complex because of rapid changes regarding climate, urbanisation, ecology, and travel frequency and the fragility of most public health systems globally. Identifying factors contributing to this problem is thus essential for ensuring its mitigation. These factors can be identified by determining infectious disease transmission modes, establishing disease prevention and control strategies, and identifying infection reservoirs.

Despite the World Health Organization issuing recommendations indicating how the re-emergence of novel virulent strains of the coronavirus can be prevented, numerous healthcare organisations remain unprepared for such events. Furthermore, countries generally employ distinct epidemic response strategies because of their unique social, historical, and cultural backgrounds and political systems. Several previous national-government-implemented epidemic response strategies may be insufficient to control outbreaks of the new Omicron variant, which may engender additional, smaller-scale COVID-19 outbreaks. Budd *et al*.^4^ reviewed digital technologies that can improve global responses to such potential smaller-scale outbreaks, indicating that epidemic prevention can be optimised by applying such technologies in public health systems to evaluate interventions, implement contact tracing, monitor the population, identify cases, and enable mobile-data-based communication with the public.

Although vaccine administration has reduced the likelihood of severe COVID-19 case development and COVID-19-related death, the SARS-CoV-2 Omicron variant’s characteristics differ from the Delta variant’s characteristics. Therefore, models for assessing disease and prevention strategies for the Delta variant were not applicable to the Omicron variant. Future research should thus explore vaccines that are effective against any variant.

In the low-income or developing countries, actively promoting vaccination and providing medical resources are essential for epidemic control. Accordingly, the World Bank established the Financial Intermediary Fund for Pandemic Prevention, Preparedness, and Response to provide long-term financing for strengthening the World Bank’s pandemic response capabilities and to address crucial problems in national, regional, and global systems for pandemic prevention and response through investment and technical support^5^. The fund can improve coordination among partners, offer complementary support, offer incentives for greater investment at the country level, provide an advocacy platform, and maintain and increase focus on improving healthcare systems.

The general perception is that the most difficult period of the COVID-19 pandemic has ended and focus should be shifted to preparing for subsequent public health emergencies. Nevertheless, the COVID-19 crisis represents a reminder that we are living in the world as a global village. All nations must engage in global preparation, collaborate in national risk assessments, provide up-to-date information regarding novel discoveries, collaborate in improving infectious disease prevention systems, assess resource adequacy for managing subsequent outbreaks, and support international scientific collaboration for studying SARS-CoV-2 to ensure sufficient action and preparedness plans for preventing pandemic re-emergence.

## Ethical approval

This is only a commentary, not research involving patients. No ethical approval is required.

## Consent

This is only a commentary, not research involving patients. No patient consent is required.

## Sources of funding

This is a commentary. We have no funding for our commentary.

## Author contribution

S.-U.F.C.: conceptualization and writing the original draft; J.-R.C.: validation; C.-C.C.: conceptualization, supervision, submission, and correspondence.

## Conflicts of interest disclosure

There are no conflicts of interest in our commentary.

## Research registration unique identifying number (UIN)

This is a commentary on a study published in ‘International Journal of Surgery’. No UIN is required.

## Guarantor

Professor Chong-Chi Chiu.

## Data availability statement

This is only a commentary on a study published in ‘International Journal of Surgery’. There is no research data in our commentary.
